# Higher Negative Self-Reference Level in Patients With Personality Disorders and Suicide Attempt(s) History During Biological Treatment for Major Depressive Disorder: Clinical Implications

**DOI:** 10.3389/fpsyg.2021.631614

**Published:** 2021-03-25

**Authors:** Samuel Bulteau, Morgane Péré, Myriam Blanchin, Emmanuel Poulet, Jérôme Brunelin, Anne Sauvaget, Véronique Sébille

**Affiliations:** ^1^UMR INSERM 1246, SPHERE MethodS in Patient-Centered Outcomes and HEalth ResEarch, University of Nantes, University of Tours, Nantes, France; ^2^CHU Nantes, Department of Addictology and Psychiatry, Nantes, France; ^3^CHU Nantes, Department of Methodology and Biostatistics, Nantes, France; ^4^INSERM-U1028, CNRS-UMR5292, Lyon Neuroscience Research Center, PSYR^2^ Team, University of Lyon, CH Le Vinatier, Lyon, France; ^5^Department of Emergency Psychiatry, Edouard Herriot Hospital, Hospices Civils de Lyon, Lyon, France; ^6^Nantes Université, CHU Nantes, Movement, Interactions, Performance (MIP), EA 4334, University of Nantes, Nantes, France

**Keywords:** major depressive disorder, venlafaxine, rTMS, patient-reported outcome, BDI, personality disorder, suicide attempt

## Abstract

**Objective:** The aim of the study was to identify clinical variables associated with changes in specific domains of self-reported depression during treatment by antidepressant and/or repetitive Transcranial Magnetic Stimulation (rTMS) in patients with Major Depressive Disorder (MDD).

**Methods:** Data from a trial involving 170 patients with MDD receiving either venlafaxine, rTMS or both were re-analyzed. Depressive symptoms were assessed each week during the 2 to 6 weeks of treatment with the 13-item Beck Depression Inventory (BDI_13_). Associations between depression changes on BDI_13_ domains (Negative Self-Reference, Sad Mood, and Performance Impairment), treatment arm, time, and clinical variables were tested in a mixed linear model.

**Results:** A significant decrease of self-reported depressive symptoms was observed over time. The main characteristics associated with persistent higher depressive symptomatology on Negative Self-Reference domain of the BDI_13_ were personality disorders (+2.1 points), a past history of suicide attempt(s) (+1.7 points), age under 65 years old (+1.5 points), and female sex (+1.1 points).

**Conclusions:** Early cognitive intervention targeting specifically negative self-referencing process could be considered during pharmacological or rTMS treatment for patients with personality disorders and past history of suicide attempt(s).

## Introduction

Major depressive disorder (MDD) is a leading cause of disability worldwide (Collins et al., 2011) and is frequently a difficult-to-treat condition causing significant burden despite usual treatment efforts (Conway et al., 2017; Bergfeld et al., 2018; McAllister-Williams et al., 2020). A poorer prognosis and relapse rate is associated with persistence of ruminations as residual symptoms (Paykel et al., 1995; Judd, 1997; Watkins and Brown, 2002). Treatment-resistance and long-lasting depressive symptomatology are associated with suicidality or comorbidities exposing to ruminations such as anxiety or personality disorders (Bennabi et al., 2015; De Carlo et al., 2016; Kautzky et al., 2017; Arditte Hall et al., 2019; Bartova et al., 2019). Lower self-esteem and suicidality can be reinforced by loneliness, impaired cognitive functioning and disability in the elderly, which is an important concern given the increasing aging of the population in many countries (Aziz and Steffens, 2013). In order to improve care of difficult-to-treat depression, the way we measure depression over time is crucial and may provide insight on how optimizing and personalizing treatment (Murphy et al., 2017). First, it is increasingly recognized that treatment goal should be to achieve full functional remission from the patients' perspective, highlighting the growing importance of focusing on patient-reported outcomes (Zimmerman et al., 2012; Sheehan, 2016; Cuijpers, 2019; Kernot et al., 2019). Second, given that depressive disorder is multidimensional, with different symptomatic expressions depending on baseline characteristics (Fried et al., 2014; Fried and Nesse, 2015), studying specific domains over time could be a more fruitful approach to guide treatment choice than only focusing on the global sum-score (that only reflects the overall psychic burden but doesn't provide any information about the underlying pathological process). Thirdly, outcomes that matter to patients include physical, emotional, autoagressive and cognitive symptoms, but also functioning and the perception of one's own self (Chevance et al., 2020). Beck depression Inventory (BDI) is the most used patient-reported outcome to assess depressive symptomatology (Beck et al., 1988). The short 13-item form of original BDI scale (Gould, 1982) is one of the simplest measure of subjective aspects of depression and widely used in daily clinical practice. The objective of this study was to analyze data from a randomized controlled trial assessing efficacy of biological treatment (rTMS, antidepressant, or a combination of both), focusing on the longitudinal self-reported changes on domains of the BDI_13_, and to identify clinical variables associated with change in domains scores over time. We first needed, as a preliminary aim, to verify the psychometric properties of the BDI_13_ in the studied population (e.g., factor structure).

## Methods

We performed a secondary analysis of data from the largest French multi-center randomized double-blind controlled trial which evaluated the efficacy of low frequency rTMS and venlafaxine in depression (Brunelin et al., 2014). A hundred and seventy patients were allocated to receive either active rTMS in combination with active venlafaxine (*n* = 55), or active rTMS and placebo venlafaxine (n = 60) or sham rTMS combined with active venlafaxine (n = 55). Patients received once-daily sessions of active or sham 1 Hz rTMS applied over the right dorsolateral prefrontal cortex (360 pulses/day delivered at 120% of the resting motor threshold; 6 trains of 60 s on separated by 30 s off) for two to six weeks; rTMS was combined with either active or placebo venlafaxine (mean dose: 179.0 +/- 36.6 mg/day). This study did not show significant difference between low frequency rTMS, venlafaxine and the combination of both treatments on the primary efficacy outcome which was the number of patients who achieved remission according to HDRS_17_ score (<8).

### Population

To be included in this study, participants had to present with a single or recurrent, unipolar MDD episode according to the DSM IV-TR. The participants were then selected if they had a HDRS_17_ score > 20 after the failure of at least one antidepressant, delivered at an efficacious dosage for at least 6 weeks. The exclusion criteria were age under 18, other axis I disorders (except for anxiety disorders) including substance use disorder (except for nicotine), somatic or neurological disorders, failure to respond to venlafaxine during the current episode, pregnancy, previous rTMS, and rTMS contraindications.

### Variables

The variables for the current analysis included: age, sex, education level, number of previous hospitalizations, age at onset of the first MDD episode, duration (months), total number of medications for the current episode, number of suicide attempt, presence of anxiety or personality disorder, family history of psychiatric disorder, and invalidity pension. Anxiety and personality disorders were declared by investigators following a clinical assessment by a psychiatrist with a lifetime perspective, based on the DSM IV-TR criteria and a structured interview using the MINI 5.0. BDI_13_ was assessed at baseline and every week during the treatment course.

### Statistical Analysis

Qualitative and quantitative variables were described as number, percentage of each category and as mean and standard deviation (SD) respectively, each week, and for each treatment arm. The distribution of missing data over time in each group was estimated with a Kaplan-Meier estimator and compared by a log-rank test. The first step of the analysis consisted in investigating the factor structure (i.e., the number and composition of domains in terms of included items) of the French version of the BDI_13_. Indeed, to our knowledge, the factor structure of the French version of the BDI_13_ has not recently been investigated in such a large sample of depressed patients. Moreover, the validity of translated questionnaires has to be evaluated as language and cultural differences may depend on cultural context (Guillemin et al., 1993). The BDI_13_ was translated into French (Delay et al., 1963) and while its psychometric properties (Collet and Cottraux, 1986; Cottraux, 1988) have already been assessed, it was more than 25 years ago on small samples of depressed patients (45 and 50 patients, respectively). Moreover, the number of domains of the BDI_13_ was debated (Beck et al., 1988). Finally, ensuring the validity of the BDI_13_ domain scores is a prerequisite for studying how they change over time. Hence, a confirmatory factor analysis was first performed on the 3-factor structure of the original version (Sad Mood, Guilt, and Performance Impairment). Good (acceptable) fit was indicated by the following criteria: root mean square error of approximation or RMSEA ≤ 0.05 (0.08), standardized root mean square residual or SRMR ≤ 0.05 (0.10) and comparative fit index or CFI ≥ 0.97 (0.95). In addition to fit assessment, reliability of the BDI_13_ domains was assessed by internal consistency using the Cronbach's alpha coefficient, α. Domains were considered reliable if α > 0.70. The confirmatory factor analysis suggested that the French version of the BDI_13_ does not satisfactorily confirm the original structure (RMSEA = 0.044, SRMR = 0.081, CFI = 0.936) and only the Performance Impairment domain was considered reliable as Cronbach alphas were equal to 0.62, 0.68, and 0.74 for Sad Mood, Guilt, and Performance Impairment domains, respectively. Hence, an exploratory factor analysis with orthogonal varimax rotation was performed.

The second step of analysis in this study consisted in fitting linear mixed models to explain changes in BDI_13_ scores throughout treatment course and identify clinical variables associated with these changes. Separate linear mixed models were fitted on each domain score of BDI_13_, identified in the first step of the analysis. Linear mixed models were chosen to analyze these longitudinal data as it is possible to characterize the mean change of the population over time with the fixed effects of the model while modeling individual variation around the mean trajectory of the score with random effects. The fixed effect part of the linear mixed model explaining each score included the treatment arm, the time, the interaction between time and treatment arm, and a set of covariates and their interaction with treatment arm at first. As little was known about the covariates associated with BDI_13_, the set of covariates was defined as the variables listed in Variables section and associated in univariate analyses with changes in BDI_13_ domain scores over time (*p* < 0.20). For each BDI_13_ score separately, the set of covariates in the fixed effect part of the linear mixed models was refined by selecting variables by backward elimination (*p* < 0.05). The final mixed model of BDI_13_ scores estimates the associations between depression changes, treatment arm and the retained variables.

Level of significance was set at 5%. Psychometric analyses were performed with Stata 15 software, and other analyses used SAS software (version 9.4, NC, USA).

### Ethic

All patients gave consent accompanied by a comprehensive assessment form. The study was registered under the ClinicalTrials.gov identifier: NCT00714090, conducted in accordance with the Declaration of Helsinki and was approved by a local ethics committee (CPP Sud est 6 #AU732).

## Results

### Factor Structure of the BDI_13_

A structure with 3 domains showed acceptable fit (RMSEA = 0.037, SRMR = 0.064, CFI = 0.954). These domains were: Sad Mood (item 1: sadness, item 2: pessimism), Negative Self-Reference (item 3: sense of failure; item 5: guilt; item 6: self-hate; item 7: suicidal ideas; item 10: negative body-image), and Performance Impairment (item 4: dissatisfaction; item 8: social withdrawal; item 9: indecision; item 11: working difficulties; item 12: fatigability). Negative Self-Reference and Performance Impairment domains showed acceptable reliability with Cronbach alpha coefficients equal to 0.73 and 0.72 respectively, contrary to the Sad Mood domain (**α** = 0.55).

### Sample Characteristics

As previously reported by Brunelin et al. (2014), the patient population (mean age of 54 +/-11 (SD) years) exhibited characteristics of severe and difficult-to-treat depression, as shown in Table 1, with: a mean HDRS_17_ 25.86 +/- 3.7; treatment resistance (>2 treatment lines on average); several previous hospitalizations (3 on average); family history of mood disorders (59%); suicidality [about 41% committed at least one suicide attempt(s)]; long lasting episode duration (mean: 19 months); severe functional impairment (48.5% had long term illness exemption); and comorbidities, especially anxiety (23.8%) or personality (30.5%) disorders. Tolerability was good and premature withdrawal due to side effects were observed in 11.1% cases in rTMS, venlafaxine and combination, overall. The distribution of missing data was not significantly different between groups (p = 0.59).

**Table 1:** Clinical and demographical characteristics of the whole sample (*N* = 170) at baseline.

	***N***	**rTMS + venlafaxine** **(*N* = 55)**	**rTMS + placebo** **(*N* = 60)**	**Venlafaxine + sham rTMS** **(*N* = 55)**	**Total** **(*N* = 170)**
		***N*** **(%) or Mean ± SD**			
Age (years)	170	54.6 ± 12.5	53.4 ± 12.1	55.2 ± 10.5	54.3 ± 11.7
Age ≥ 65 years	170	10 (18.2%)	10 (16.7%)	8 (14.6%)	28 (16.5%)
Female	169	36 (66.7%)	38 (63.3%)	37 (67.3%)	111 (65.7%)
Number of hospitalizations	164	3.2 ± 4.4	3.5 ± 5.9	2.3 ± 2.5	3.0 ± 4.5
Age at first depression onset (years)	167	40.1 ± 14.5	40.7 ± 13.5	37.7 ± 12.5	39.5 ± 13.5
Episode duration (months)	165	15.9 ± 18.0	14.6 ± 15.5	27.1 ± 49.6	19.0 ± 31.6
Episode duration ≥ 2 years	165	14 (26.4%)	15 (25.4%)	18 (34.0%)	47 (28.5%)
Number of previous treatment lines	163	2.8 ± 2.2	2.4 ± 1.9	2.7 ± 1.7	2.6 ± 1.9
Anxiety disorder	168	13 (24.5%)	14 (23.3%)	13 (23.6%)	40 (23.8%)
Personality disorder type:	170	13 (26.0%)	19 (32.2%)	18 (32.7%)	50 (30.5%)
Missing data		4	6	5	15
Histrionic		2 (22.2%)	5 (38.5%)	4 (30.77)	11 (31.4%)
Dependant		2 (22.2%)	2 (15.4%)	1 (7.7%)	8 (22.9%)
Borderline		2 (22.2%)	4 (30.8%)	1 (7.7%)	7 (20.0%)
Narcissic		0 (0.0%)	1 (7.7%)	1 (7.7%)	2 (5.7%)
Obsessive-compulsive		0 (0.0%)	0 (0.0%)	1 (7.7%)	1 (2.9%)
Non specified		3 (33.3%)	1 (7.7%)	2 (15.4%)	6 (17.1%)
Family history of psychiatric disorders	168	36 (66.7%)	36 (60.0%)	27 (50.0%)	99 (58.9%)
Family history at the first degree of psychiatric disorders	168	35 (63.6%)	32 (53.3%)	22 (40.0%)	89 (53.0%)
History of suicide attempt(s)	164	23 (44.2%)	25 (41.7%)	19 (36.5%)	67 (40.8%)
Long-term illness exemption	169	27 (50.0%)	33 (55.0%)	22 (40.0%)	82 (48.5%)
Disability pension for depression	169	7 (13.0%)	12 (20.0%)	7 (12.7%)	26 (15.4%)
BDI_13_ Negative Self-Reference	165	6.7 ± 3.5	7.6 ± 3.9	7.0 ± 3.3	7.1 ± 3.6
BDI_13_ Sad Mood	166	3.7 ± 1.5	3.7 ± 1.5	3.7 ± 1.5	3.7 ± 1.5
BDI_13_ Performance Impairment	166	8.8 ± 2.1	9.1 ± 2.7	9.2 ± 2.8	9.0 ± 2.5
HDRS21 total score	168	27.5 ± 4.5	26.9 ± 3.9	27.0 ± 3.9	27.1 ± 4.1

### Variables Associated With Longitudinal Change in Self-Reported Depression Domains Scores

After 6 weeks of treatment, we observed in the whole sample a significant decrease of depression scores with a mean decrease of−0.6,−0.3,−0.6 points per week on average for the Negative Self-Reference, Sad Mood, and Performance Impairment domains of the BDI_13_, respectively.

Treatment groups were not significantly associated with changes in any domains of the BDI_13_. Patients' characteristics significantly associated with higher mean scores of Negative Self-Reference at baseline and during treatment course were: personality disorder (+2.1 points) or past history of suicide attempt (+1.7 points), age under 65 years (+1.5) or female (+ 1.1 points) as presented in Table 2.

**Table 2:** Estimates of the fixed effects of linear mixed models with correlated intercept and random slope for the 3 domains of the BDI_13_, *N* = 170.

**Scale**	**Variable**	**Estimate**	**95% CI**	***p*-value**
BDI Negative Self-Reference	Intercept	5.6	[4.6; 6.7]	<0.0001
	Treatment arm			0.1842
	rTMS + venlafaxine placebo	1.0	[-0.1;0 2.1]	0.0742
	rTMS + venlafaxine	0.3	[-0.8; 1.5]	0.5983
	Venlafaxine + sham rTMS (ref)	0		
	Time (+1 week)	−0.6	[-0.7;−0.5]	<0.0001
	Personality disorder	2.1	[1.1; 3.1]	<0.0001
	History of suicide attempt	1.7	[0.7; 2.7]	0.0005
	Age ≥ 65	−1.5	[-2.7;−0.3]	0.0189
	Male	−1.1	[-2.0;−0.1]	0.0312
BDI Sad Mood	Intercept	3.1	[2.7; 3.5]	<0.0001
	Treatment arm			0.4222
	rTMS + venlafaxine placebo	0.3	[-0.2; 0.7]	0.3015
	rTMS + venlafaxine	0.3	[-0.2; 0.8]	0.2212
	Venlafaxine + sham rTMS (ref)	0		
	Time (+1 week)	−0.3	[-0.4;−0.3]	<0.0001
BDI Performance impairment	Intercept	8.5	[7.9; 9.1]	<0.0001
	Treatment arm			0.8504
	rTMS + venlafaxine placebo	0.2	[-0.7; 1.1]	0.6803
	rTMS + venlafaxine	−0.1	[-1.0; 0.9]	0.8979
	Venlafaxine + sham rTMS (ref)			
	Time (+1 week)	−0.6	[-0.8;−0.5]	<0.0001

*BDI_13_, Beck Depression Inventory 13 items; IC, confidence interval*.

**Figure 1:**
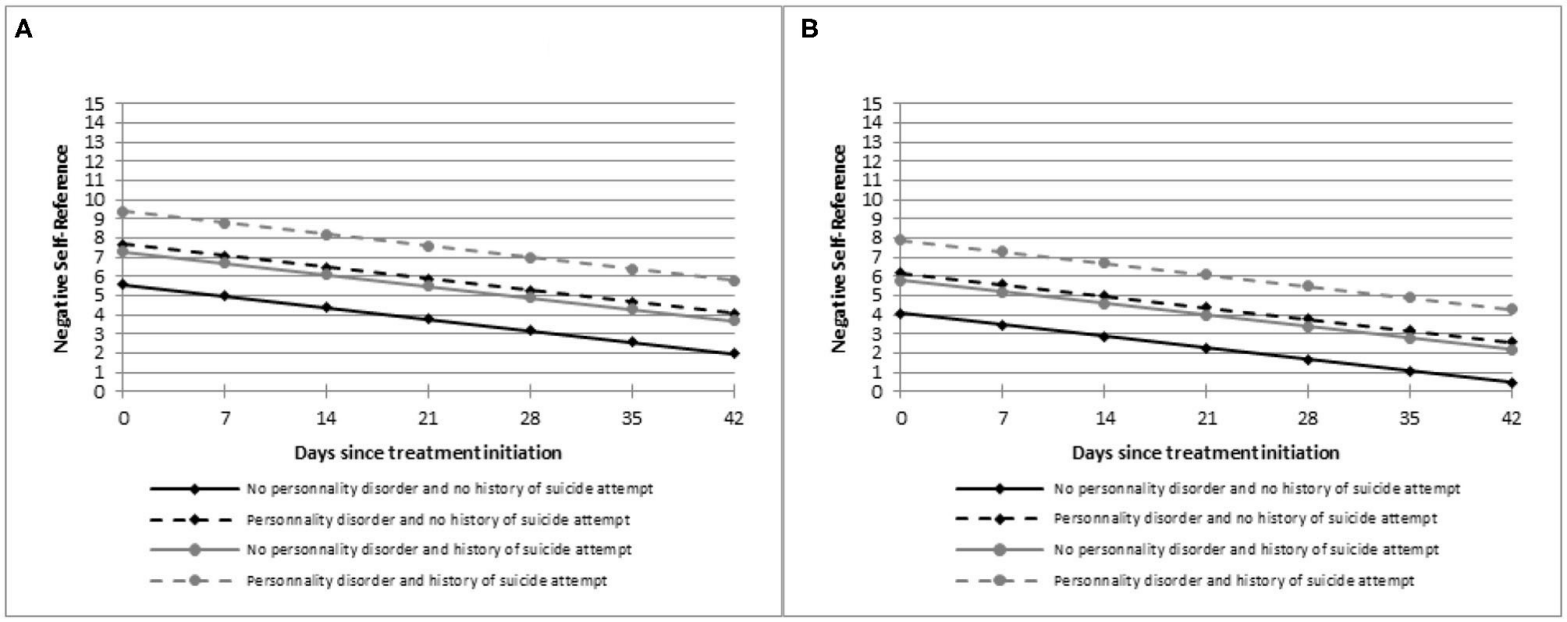
Estimated means of negative self-reference scores over time according to age, personality disorders, and history of suicide attempts. Only means for women (reference category of the linear mixed model) are represented. **(A)** patients under 65 years. **(B)** patients aged 65 years or older.

Specifically, the mean score of the Negative Self-Reference domain was the highest at baseline and also during treatment course for women under 65 years with personality disorder and a history of suicide attempt(s) as compared to other patients. The effects of the most significant covariates, namely personality disorder, past history of suicide attempt and age, on the estimated means of Negative Self-Reference score over time from the linear mixed model are represented on Figure 1. On Figure 1A, women under 65 years old with no personality disorder nor history of suicide attempt have a mean score of Negative Self-Reference of 5.6 points (intercept estimate, Table 2) at baseline and of 2 points after 6 weeks of treatment (decrease of depression:−0.6 points per week, *p* < 0.05) as represented with a solid black line. The decrease of depression is similar for women under 65 years old with personality disorder and a history of suicide attempt (Figure 1A, dotted gray line) but they have on average higher scores of Negative Self-Reference at baseline, i.e., 9.4 points (intercept +2.1 points for personality disorder and +1.7 points for history of suicide attempt, Table 2) and after 6 weeks of treatment, i.e., 5.8 points. Hence, their levels of Negative Self-Reference are higher at baseline and also remain higher during the 6-week follow-up. The same trends are observed for patients aged 65 years or older (Figure 1B) with significantly lower mean scores (age estimate:−1.5 points, Table 2). The effect of age can be observed by comparing the curves with the same layout between Figures 1A,B, for example the solid black lines for women with no personality disorder nor history of suicide attempt. None of the variables, except time, were associated with Performance Impairment and Sad Mood domain scores at baseline and over time.

## Discussion

### Main Findings

To the best of our knowledge, this is the first study focusing on longitudinal changes in self-reported depressive domains including negative self-reference and identifying clinical variables associated with these changes. It was a secondary analysis of the largest clinical trial including both rTMS and antidepressant treatments (Brunelin et al., 2014).

First, we described a 3-factor structure of the BDI_13_ (i.e., Negative Self-Reference, Sad Mood and Performance Impairment) showing acceptable fit. More specifically, Negative Self-Reference mainly dealt with negative self-regard (feeling of failure; guilt; self-disgust; negative self-image; suicidal tendencies). Similar domains, i.e.: “Negative Attitude Toward Self” and “Performance Impairment,” were also reported for the BDI-II scale (Shafer, 2006; Wang and Gorenstein, 2013).

Second, previous suicide attempt(s) and personality disorders were associated with higher depression scores at baseline and during treatment course for the Negative Self-Reference domain of the BDI_13_, regardless of treatment arm.

These higher scores are clinically meaningful considering that full remission from the patient point of view require the restoration of self-confidence and a “normalization” of self-perception (Zimmerman et al., 2012). Moreover, Negative Self-Reference is a key prognostic dimension since resistance to antidepressants is associated with excessive self-focus, negative self-regard, cognitive inflexibility, hopelessness, feeling of inadequacy, and suicidal tendencies (Mor and Winquist, 2002; Nolen-Hoeksema et al., 2008; Zahn et al., 2015; Kneeland et al., 2016; Cândea and Szentagotai-Tătar, 2018; Huprich, 2019; Rush et al., 2019). A woman under 65 years old and suffering from personality disorder and past suicide attempt(s) history can have up to 6.4 additional points on Negative Self Reference domain compared to older male patients without these antecedents, so that this subgroup is more likely to present a sustained higher qualification of episode severity, for example from a mild (4-7) to moderate (8-15), or from moderate to severe (16 and above). Thus, monitoring negative self-regard during treatment course is an important clinical issue.

### Association Between Suicidality and Negative Self-Reference Domain

We observed that suicide attempters had higher level of negative self-reference, which is consistent with the literature. Suicidal behaviors and negative self-reference seem to have a bidirectional link: self-contempt may be a risk factor for suicidality and suicidal behaviors lead to negative attitude toward oneself (Jollant et al., 2017; Rüsch et al., 2019; Solano et al., 2019). Moreover, recent findings based on the assessment of functional connectivity between neuronal networks, showed particular patterns of activation of the Default-Mode Network (DMN) supporting self-referencing process, in suicide attempters vs. non-attempters suffering from MDD, and healthy controls. Authors suggested that suicidal behavior may be due in part to impaired self-referential thought processing and alters cognitive flexibility supported by the Central Executive Network (CEN) (Malhi et al., 2020). This is consistent with the description in MDD of an imbalance between an increased resting state activity in the DMN (self-focus) (Nejad et al., 2013), and a hypo-activity of the CEN (supporting executive functioning) (Boeker and Kraehenmann, 2018). In summary, our findings confirm the importance to measure self-reference domain in the follow-up of suicide attempters' follow up.

### Association Between Personality Disorders and Negative Self-Reference Domain

Personality disorders are known to be a risk factor for MDD (Bennabi et al., 2015; De Carlo et al., 2016; Kautzky et al., 2017; Roselli et al., 2020). They expose to negative affectivity and negative self-regard making it difficult to disentangle the two diagnoses during a depressive episode. Symptoms explained by the depressive state are more likely to change during treatment than those related to temperament or personality. Studies investigated the role of temperament (mainly using the Cloninger Temperament Inventory) and highlighted the importance of two domains -High Harm Avoidance (reflecting negative affectivity) and Low Self-Directedness- as trait markers associated with MDD (Zaninotto et al., 2016), as well as suicidality (Erić et al., 2017).

Trait malignant self-regard can explain higher level of depression and poorer outcome. However, little is known specifically about the links between personality disorders and self-reference process. Most of published studies focused on borderline disorders which is not the most frequent personality disorder associated with TRD. Moreover, physiopathology of negative self-referencing process has been little investigated, especially how the dorsomedial prefrontal cortex integrates increased attention to negative stimuli with an increased attention to the self in patients with personality disorders and MDD vs. MDD alone (Lemogne et al., 2011).

In summary, Negative Self-Reference may be related to both MDD state and personality traits, and patients with personality disorders are more likely to remain impaired on that clinical dimension.

### Consequences for Clinicians and Treatments

Our findings highlight the value of focusing on domains rather than only on a global score given that depression may not be a homogeneous syndrome (Fried and Nesse, 2015). The impact of personality disorder and/or previous suicide attempts (representing 30.5 and 41% patients in our sample, respectively) on the Negative Self-Reference domain may draw the attention on the influence, from the patient's perspective, of those two variables on MDD prognosis whatever biological treatment type (brain stimulation and antidepressants). Our findings highlight the interest to consider interventions targeting negative self-regard in patients with personality disorder or past history of suicidal attempts. In line with this, Cognitive Behavioral Therapy may be efficacious for treating individuals with low self-esteem, according to changes in self-reported measures (Kolubinski et al., 2018). Several other psychotherapeutic approaches targeting self-representations and reappraisal, like psycho-education or cognitive remediation, are useful in addition to biological treatments (Pandarakalam, 2018).

Identifying such clinical factors associated with Negative Self-Reference pave the way for concomitant synergistic adjuvant interventions that may be promising throughout their ability to reduce ruminations, negative self-image, and improve cognitive capacities as well as perceived self-efficacy (Donse et al., 2018; Neacsiu et al., 2018; Laird et al., 2019; Wilkinson et al., 2019). Surprisingly, anxiety disorders were not significantly associated with the negative self-reference domain, despite its prevalence in TRD (Nuñez et al., 2018). We could have expected also higher negative self-referencing process in patients with baseline Generalized anxiety (GAD) since rumination, which frequently consists in negative self-judgment, is a core feature of both GAD and MDD (Merino et al., 2016). One can hypothesize that GAD was more associated with other features not captured by the BDI_13_, such as worry about life circumstances, anxious apprehension of events or cognitive bias toward potential threat (Crocq, 2017). Contrariwise to our expectation that older age would be associated with higher depressive symptoms level, patients older than 65 years demonstrated lower Negative Self-Reference domain score. This could be explained by a positive cognitive bias and reappraisal of symptoms, which has been described in aging (Orth et al., 2010; Whitehead and Bergeman, 2016).

## Limitations

Although being one of the largest French study investigating medications and rTMS in MDD, sample size and power remain limited. Moreover, attrition was quite high, although its rate was not significantly different between groups. The proposed 3-factor structure of the BDI showed an acceptable fit but the Sad Mood domain showed quite low reliability reflected by a Cronbach's alpha <0.7; this may have been due to the small number of items included in this domain affecting reliability. The assessment of psychometric properties of the French version of the BDI-13 was incomplete as data come from a clinical trial not designed for a validation study. As the literature about the validity and reliability of the French version of the BDI-13 remains scarce, its psychometric properties have to be fully assessed in the future in a specifically designed study with a large sample size. As adjustments for multiple testing was not done in this exploratory study, we cannot exclude chance findings. However, several authors have suggested that adjustment for multiple comparisons may not be desirable for exploratory studies as it may, for instance, increase the type II error (Rothman, 1990). But of course, this also implies that the associations that were evidenced need to be confirmed in subsequent confirmatory studies. Conducting an exploratory analysis may be seen as a limitation but allowed us to gain insight into the data and identify the most influential patients' characteristics without an *a priori*. Finally, no specific questionnaire such as Structured Clinical Interview for DSM (SCID) interview has been used to diagnose personality disorder. This diagnosis was however assessed carefully by trained psychiatrists, experts in mood disorder evaluation, and according to the patient anamnesis and DSM IV-TR criteria. Lastly, we can mention that patients with similar health outcomes may not rate self-reported data in the same way over time, since the understanding or interpretation of items may change. This change in the meaning of one's self-evaluation of a construct over time is known as Response Shift (RS) (Schwartz and Sprangers, 1999). RS results from change in the respondent's internal standard of measurement (recalibration), in the importance of component domains constituting the target construct (reprioritization), or a redefinition of the construct itself (reconceptualization). RS may lead to biased estimations of treatment effects based on PRO. Thus, the identification of RS warrants further investigation in psychiatry (Bulteau et al., 2019).

## Conclusion

To conclude, personality disorders and previous suicide attempts were the two main factors associated with worsened self-reported symptomatology within the negative self-reference domain, at baseline and during treatment course in a sample of patients suffering from difficult-to-treat depression. Those results may draw the attention of clinicians to the interest of synergistic interventions targeting specifically negative self-referential process in addition to rTMS and/or drugs for patients presenting with personality disorder and/or past history of suicide attempts.

## Data Availability Statement

The data that support the findings of this study are available from the corresponding author upon reasonable request. Requests to access these datasets should be directed to jerome.brunelin@ch-le-vinatier.fr.

## Ethics Statement

The studies involving human participants were reviewed and approved by CPP Sud Est 6 #AU732. The patients/participants provided their written informed consent to participate in this study.

## Author Contributions

SB, VS, MB, and AS wrote the manuscript. VS, MB, and MP were responsible for performing statistical analyses. EP and JB were the main investigators of the princeps study and contributed for database use and manuscript improvement. All authors reread, gave significant contribution and comments and approved the final manuscript.

## Conflict of Interest

The authors declare that the research was conducted in the absence of any commercial or financial relationships that could be construed as a potential conflict of interest.
